# Organic Cation Transporter-Mediated Ergothioneine Uptake in Mouse Neural Progenitor Cells Suppresses Proliferation and Promotes Differentiation into Neurons

**DOI:** 10.1371/journal.pone.0089434

**Published:** 2014-02-25

**Authors:** Takahiro Ishimoto, Noritaka Nakamichi, Hiroshi Hosotani, Yusuke Masuo, Tomoko Sugiura, Yukio Kato

**Affiliations:** Faculty of Pharmacy, Institute of Medical, Pharmaceutical and Health Sciences, Kanazawa University, Kanazawa, Ishikawa, Japan; University of Iowa, United States of America

## Abstract

The aim of the present study is to clarify the functional expression and physiological role in neural progenitor cells (NPCs) of carnitine/organic cation transporter OCTN1/SLC22A4, which accepts the naturally occurring food-derived antioxidant ergothioneine (ERGO) as a substrate *in vivo*. Real-time PCR analysis revealed that mRNA expression of OCTN1 was much higher than that of other organic cation transporters in mouse cultured cortical NPCs. Immunocytochemical analysis showed colocalization of OCTN1 with the NPC marker nestin in cultured NPCs and mouse embryonic carcinoma P19 cells differentiated into neural progenitor-like cells (P19-NPCs). These cells exhibited time-dependent [^3^H]ERGO uptake. These results demonstrate that OCTN1 is functionally expressed in murine NPCs. Cultured NPCs and P19-NPCs formed neurospheres from clusters of proliferating cells in a culture time-dependent manner. Exposure of cultured NPCs to ERGO or other antioxidants (edaravone and ascorbic acid) led to a significant decrease in the area of neurospheres with concomitant elimination of intracellular reactive oxygen species. Transfection of P19-NPCs with small interfering RNA for OCTN1 markedly promoted formation of neurospheres with a concomitant decrease of [^3^H]ERGO uptake. On the other hand, exposure of cultured NPCs to ERGO markedly increased the number of cells immunoreactive for the neuronal marker βIII-tubulin, but decreased the number immunoreactive for the astroglial marker glial fibrillary acidic protein (GFAP), with concomitant up-regulation of neuronal differentiation activator gene Math1. Interestingly, edaravone and ascorbic acid did not affect such differentiation of NPCs, in contrast to the case of proliferation. Knockdown of OCTN1 increased the number of cells immunoreactive for GFAP, but decreased the number immunoreactive for βIII-tubulin, with concomitant down-regulation of Math1 in P19-NPCs. Thus, OCTN1-mediated uptake of ERGO in NPCs inhibits cellular proliferation via regulation of oxidative stress, and also promotes cellular differentiation by modulating the expression of basic helix-loop-helix transcription factors via an unidentified mechanism different from antioxidant action.

## Introduction

Neural progenitor cells (NPCs) have self-renewal ability, as well as pluripotentiality to differentiate into neurons, astrocytes and oligodendrocytes [1], [2]. Owing to the ability, NPCs can occur neurogenesis even in the adult mammalian brain [3], [4] in spite of the fact that severely damaged neurons cannot regenerate [5], [6]. Focusing on the differentiation into neurons, NPCs proliferate in an undifferentiated state, followed by differentiation into immature neurons and subsequent growth to mature neurons [7], [8]. The ability of NPCs to proliferate or differentiate into neurons is regulated by extracellular signal regulatory molecules, neurotransmitter receptors and transporters expressed on cellular membranes. It has been reported that various neurotransmitter receptors and membrane transporters are expressed in NPCs [9]. However, extracellular signal regulatory molecules involved in the physiological regulation of NPCs *in vivo* have not been fully clarified. Physiological control by neurotransmitter receptors must be important in mature neurons, which form many synapses. However, regulation by neurotransmitter receptors expressed in NPCs may not be so important *in vivo*, because they are immature and undifferentiated cells that do not form synapses. On the other hand, transporters control membrane permeation of their substrates, thereby regulating the intracellular concentrations of important molecules. Therefore, in the present study we have focused on the possible role of membrane transporters in regulating differentiation and/or proliferation of NPCs.

Transporters have been categorized into two superfamilies, ATP-binding cassette (ABC) and solute carrier (SLC) transporters. ABC transporters exclude substrates from the intracellular space, whereas SLC transporters are involved in both influx and efflux of their substrates. ABCB1 and ABCG2 are expressed in human NPCs [10], [11]. The number of sex determining region Y-box 2 (Sox2)-positive NPCs was significantly increased in *abcc1*-deficient mice, while there was no effect on NPCs in *abcb1*- or *abcg2*-deficient mice [12]. Early doublecortin-positive neurons were significantly decreased in the absence of *abcb1* and *abcg2*, while later post-mitotic calretinin-positive cells were reduced only in *abcb1* transporter-deficient mice [12]. Thus, ABCB1, ABCC1 and ABCG2 differentially regulate neuroregeneration [12]. SLC transporters, on the other hand, have been divided into so-called physiological transporters that are involved in various biological events through their ability to recognize neurotransmitters, including dopamine, serotonin and glutamic acid, or their precursor substances, as substrates, and xenobiotic transporters that have relatively broad substrate specificity. Excitatory amino acid transporters 1 and 2 negatively regulate calcium-dependent proliferation of hippocampal NPCs and are persistently up-regulated after injury [13]. Glutamine transporter positively regulates proliferation and neuronal differentiation in neural progenitor model P19 cells [14]. These reports mostly focused on the roles of ABC and physiological SLC transporters in regulation of NPCs, but the roles of the xenobiotic SLC transporters in NPCs are less well understood.

Carnitine/organic cation transporter OCTN1/SLC22A4, classified as a xenobiotic transporter, is ubiquitously expressed in the body [15]–[17], and transports various therapeutic agents, including organic cations and zwitterions [15], [18]–[24]. We recently succeeded in producing *octn1* gene knockout (*octn1^−/−^*) mice and found by means of metabolome analysis that the naturally occurring antioxidant ergothioneine (ERGO) is a good *in vivo* substrate of OCTN1 [25]. OCTN1 is functionally expressed in mouse small intestine, liver, kidney and brain [25]–[27]. In the brain, OCTN1 is functionally expressed in neurons [27], but not astrocytes or vascular endothelial cells [28], [29]. However, it has not yet been clarified whether OCTN1 is functionally expressed in NPCs. We have previously demonstrated that OCTN1 suppresses proliferation and promotes differentiation of mouse neuroblastoma Neuro2a cells, which exhibit some characteristics of neuronal progenitor cells, i.e. are determined to differentiate into neuron and do not have pluripotentiality [27], but no information is available on possible expression and function of OCTN1 in NPCs.

The aim of the present study is to clarify the physiological relevance of OCTN1-mediated ERGO uptake in NPCs. First, to confirm functional expression of OCTN1 and its contribution to ERGO uptake in NPCs, we examined expression of OCTN1 at the level of mRNA and protein in mouse cultured cortical NPCs, and compared uptake of [^3^H]ERGO in NPCs between *octn1^+/+^* (wild-type) and *octn1^−/−^* embryonic mice. To examine the physiological meaning of OCTN1-mediated ERGO uptake in NPCs, we investigated the biological effect of the OCTN1 substrate ERGO on proliferation and differentiation of cultured NPCs by adding ERGO to the culture medium. Furthermore, we examined the effect of knockdown of OCTN1 on proliferation and differentiation ability in NPCs-model mouse embryonic carcinoma P19 cells by treatment with siRNA for OCTN1.

## Materials and Methods

### Materials

[^3^H]ERGO (1 Ci/mmol) and [^14^C]mannitol (55 mCi/mmol) were purchased from Moravek Biochemicals (Brea, CA, USA). Clearsol I was obtained from Nacalai Tesque (Kyoto, Japan). L−(+)−Ergothioneine-d9 was purchased from Toronto Research Chemicals, Inc. (North York, Toronto, Canada). Dulbecco's modified Eagle's medium (DMEM), DMEM/Nutrient Mixture F-12 Ham (DMEM/F12), poly-L-lysine, all-*trans* retinoic acid (ATRA), monoclonal antibodies against microtubule-associated protein 2 (MAP2), βIII-tubulin, and glial fibrillary acidic protein (GFAP) were purchased from Sigma-Aldrich (St. Louis, MO, USA). Monoclonal antibody against Na^+^/K^+^-ATPase was provided by Santa Cruz Biotechnology, Inc. (Santa Cruz, CA, USA). Recombinant human basic FGF and recombinant human EGF were provided by Pepro Tech (Rocky Hill, New Jersey, USA). FBS was supplied by Biowest (Nuaillé, France). Antiserum against the carboxyl terminus of mouse OCTN1 was produced in our laboratory [26]. Secondary antibodies conjugated with Alexa Fluor series, Lipofectamine RNAiMAX, Opti-MEM, small interfering RNA (siRNA) targeting the mouse OCTN1 gene (siOCTN1), and non-targeting (negative control) siRNA were provided by Invitrogen (San Diego, CA, USA). ISOGEN was purchased from Nippon Gene (Tokyo, Japan). MultiScribe Reverse Transcriptase was obtained from Applied Biosystems (Foster City, CA, USA). THUNDERBIRD SYBR qPCR Mix and Can Get Signal were purchased from TOYOBO (Osaka, Japan). Mouse embryonic carcinoma P19 cells were supplied by ATCC (Manassas, VA, USA). Block Ace was provided by DS Pharma Biomedical (Suita, Japan). All other chemicals and reagents were of the highest purity available and were purchased from commercial sources.

### Preparation of neural progenitor cells

Pregnant ICR mice for neural progenitor cell culture were purchased from Japan SLC (Hamamatsu, Japan). The *octn1^−/−^* mice were generated according to the previous report [25], and were backcrossed to C57BL/6J strain. In brief, the chimeric mice were cross-bred with C57BL/6J wild-type mice to obtain heterozygous animals. Heterozygous animals with 6 backcross generations into C57BL/6J were bred to generate wild-type and knockout littermates. The mice were kept in a temperature- and light-controlled environment with standard food and tap water provided *ad libitum*. This study was carried out in strict accordance with the recommendations in the Guide for the Care and Use of Laboratory Animals of the Japanese Society for Pharmacology. The protocol was approved by the Committee on the Ethics of Animal Experiments of the University of Kanazawa (Permit Number: 112047) with an effort to minimize the number of animals used and their suffering.

Cortical neural progenitor cell culture was carried out according to the method of Yoneyama et al. (2010) [30], with minor modifications. In brief, cerebral cortices from 15-day-old embryonic ICR mice, wild-type or *octn1^−/−^* C57BL/6J mice were dissected and incubated with 0.25% trypsin in phosphate-buffered saline (PBS) containing 28 mM glucose at 37°C for 20 min. Cells were mechanically dissociated by using a 1,000 μL pipette tip in culture medium and plated at a density of 5×10^5^ cells/mL on 0.2% agarose-coated 6-well dishes for culture under floating conditions. First, cortical neural progenitor cells were cultured in DMEM/F12 supplemented with 100 U/mL penicillin, 100 μg/mL streptomycin, 100 μg/mL apo-transferrin, 20 nM progesterone, 5.2 ng/mL sodium selenite and 60 μM putrescine, 10 ng/mL EGF, and 10 ng/mL basic FGF for 6 days at 37°C in a humidified 5% CO_2_ incubator. They were cultured for a period up to 6 days *in vitro* (DIV) in the growth medium with a half medium change at 3 DIV as primary cultures of NPCs. Neurospheres were formed in a culture period-dependent manner from clusters of proliferating cells under floating culture conditions, and were transferred to non-agarose-coated 6-well dishes at 3 DIV. Neurospheres in the 6 DIV primary cultures were dispersed by using a Neuro Cult Chemical Dissociation Kit (Stem Cell Technologies Inc., Cambridge, UK), and then replated at a density of 1×10^5^ cells/mL on 6-well or 24-well dishes as secondary cultures in either the presence or absence of antioxidants. The cells were kept in the growth medium for various times up to 9 DIV under the same conditions as described for the primary cultures. A half medium change was performed every 3 days. Experiments in the present study were performed using the secondary cultures.

Neurospheres cultured for 9 DIV in the secondary cultures were dispersed by using a Neuro Cult Chemical Dissociation Kit, followed by seeding at a density of 3×10^5^ cells/mL on 4-well dishes previously coated with poly-L-lysine for culture under adhesion conditions and subsequent culture was conducted in the absence of growth factors for an additional 6 days in DMEM supplemented with 5% FBS, 100 U/mL penicillin, 100 μg/mL streptomycin, 28 mM glucose, 2 mM glutamine, 5 mM HEPES, 25 µg/mL apo-transferrin, 250 ng/mL insulin, 0.5 pM β-estradiol, 1.5 nM triiodothyronine, 10 nM progesterone, 4 ng/mL sodium selenite and 50 μM putrescine with a medium change at 3 days. Removal of growth factors led to the complete abolition of nestin immunoreactivity along with a drastic increase in the number of cells immunoreactive for either MAP2 or GFAP on 6 DIV [14].

### Culture of P19 cells

Pluripotent P19 stem cells derived from murine embryonal carcinoma were cultured according to the method of Nakamura et al [31], with minor modifications. P19 cells were plated at a density of 1×10^5^ cells/mL on 0.2% agarose-coated 6-well dishes in alpha minimal essential medium (αMEM) supplemented with 100 U/mL penicillin, 100 μg/mL streptomycin, 5% FBS and 0.5 µM ATRA to differentiate into neural progenitor-like cells (P19-NPCs), followed by culture for 3 days under floating conditions as primary cultures of P19-NPCs. Neurospheres were formed from clusters of proliferating cells in a culture period-dependent manner under these conditions. Neurospheres in the 3 DIV primary cultures were dispersed with 0.25% trypsin in PBS containing 0.03% EDTA at 37°C for 2 min, and then transiently transfected with siOCTN1 or negative control siRNA by using lipofectamine RNAiMAX in Opti-MEM according to the manufacturer's instructions. The cells were replated at a density of 1×10^5^ cells/mL on agarose gel-coated 6-well dishes as secondary cultures. Neurospheres in the 3 DIV secondary cultures were dispersed with trypsin–EDTA, followed by seeding at a density of 3×10^5^ cells/mL on 4-well dishes previously coated with poly-L-lysine for culture under adhesion conditions, then cultured for 8 DIV in αMEM supplemented with 100 U/mL penicillin, 100 μg/mL streptomycin, 10% FBS with a medium change at 4 days.

### Western blotting

Membrane fractions were prepared according to the method of Khunweeraphong et al [32], with minor modifications. For crude membrane preparation, cultured NPCs were collected and suspended in 4 mL of buffer containing 50 mM Tris-HCl (pH 7.4), 150 mM NaCl, 5 mM EDTA, 1 mM phenylmethylsulfonyl fluoride, 1 mM Na_3_VO_4_ and 1 mM NaF. Cells were then disrupted in a Dounce tissue grinder 100 times on ice. Cell debris was separated by centrifugation at 1,000 g for 5 min at 4°C. The crude membrane supernatant was collected and centrifuged at 45,000 g for 30 min at 4°C. The pellet was suspended in 50 mM Tris-HCl (pH 7.4), 75 mM NaCl, 1 mM EDTA, 1 mM phenylmethylsulfonyl fluoride, and 1% NP-40. Protein concentration was determined with a Bio-Rad Protein Assay Kit.

Western blot analysis was carried out according to the method of Nakamichi et al [33], with minor modifications. Membrane suspensions were added at a volume ratio of 4∶1 to 10 mM Tris–HCl buffer (pH 6.8) containing 10% glycerol, 2% sodium dodecylsulfate, 0.01% bromophenol blue, and 5% mercaptoethanol. Each aliquot of 5 μg proteins was loaded on a 10% polyacrylamide gel for electrophoresis at a constant current of 21 mA/plate for 30 min at room temperature using a compact-slab size PAGE system (ATTO, Tokyo, Japan), followed by blotting to a polyvinylidene fluoride membrane previously treated with 100% methanol. After blocking with Block Ace solution, the membrane was reacted with antiserum against OCTN1 (1∶100) or anti-Na^+^/K^+^-ATPase antibody (1∶2000) diluted with Can Get Signal (TOYOBO, Osaka, Japan), followed by reaction with anti-mouse IgG (1∶10000) or anti-rabbit IgG (1∶100000) antibody conjugated with peroxidase. Proteins that reacted with those antibodies were detected with the aid of ECL detection reagents using a Lumino image analyzer (LAS-4000; FUJIFILM, Tokyo, Japan). Densitometric analysis was carried out using ImageJ software.

### MTT assay

Mitochondrial activity as an index of cell proliferation was determined by using the MTT assay according to the method of Yoneyama et al [30], with minor modifications. In brief, MTT solution (0.5 mg/mL in PBS) was added to each well of the culture dishes, and then the cells were incubated for 1 h at 37°C. Subsequently, solubilizing solution (0.04 M HCl in isopropanol) at equivalent volume to the MTT solution was added, and the absorbance at 570 nm was measured.

### LDH assay

Cytotoxicity was determined by measuring LDH released into the medium [34]. LDH assay was carried out according to the method of Abe et al [35], with minor modifications. In brief, NPCs and medium were separated by centrifugation at 1,000 g for 5 min at 4°C. After the centrifugation, 50 µL aliquots of the supernatant were distributed in a 96-well dish and incubated with an equal volume of reaction mixture (2.5 mg/mL L-lactate lithium, 2.5 mg/mL β-nicotinamide adenine dinucleotide, 600 μM MTT, 100 μM 1-methoxyphenazine methosulfate diluted with 200 mM Tris-HCl (pH 8.2)) for 15 min at 37°C. On the other hand, separated NPCs were washed once with PBS and solubilized in 0.2% Triton X-100 in PBS (same volume as the culture medium: 500 µL) for 10 min at 25°C. The unsolubilized fraction was separated by centrifugation at 16,400 g for 5 min at 4°C. After the centrifugation, the supernatant was collected and incubated with the reaction mixture as described above. Finally, the reaction was stopped by adding 100 µL of stop buffer (50% DMF/20% SDS, pH 4.7). The absorbance was measured with a microplate reader at a test wavelength of 570 nm and a reference wavelength of 655 nm.

### Intracellular ROS imaging

Intracellular ROS imaging was performed according to the method of Yoneyama et al [30], with minor modifications. In brief, cells were incubated with 10 µM ROS-reactive fluorescent reagent CM-H_2_DCFDA for 1 h at 37°C, followed by observation under a confocal laser scanning microscope (LSM710; Carl Zeiss, Jena, Germany). The population of neurospheres showing strong intensity was measured by using ImageJ software.

### Quantitative RT-PCR

Total RNA was extracted from cultured cells according to the standard ISOGEN procedure. cDNA was synthesized with oligo (dT)_12–18_ primer, deoxynucleotide triphosphate mix, RT buffer and MultiScribe Reverse Transcriptase and amplified on a Mx3005P (Agilent Technologies; Santa Clara, CA, USA) in a reaction mixture containing cDNA with relevant sense and antisense primers (Table 1), and THUNDERBIRD SYBR qPCR Mix. PCR reactions were initiated by template denaturation at 95°C for 15 min, followed by 40 cycles of amplification (denaturation at 95°C for 10 s, and primer annealing and extension at 60°C for 30 s). The expression levels of mRNA were normalized to an internal standard, acidic ribosomal phosphoprotein P0 (36B4) or glyceraldehyde-3-phosphate dehydrogenase (GAPDH). PCR efficiency varies depending on the primer, and an absolute quantification method is needed for comparing absolute amounts of several mRNAs. Using several diluted cDNAs, a calibration curve of absorption against concentration was prepared, and absolute expression of mRNA was measured. Each cDNA was diluted with 10 mM Tris-HCl buffer (pH 8.0) containing 1 ng/µL herring sperm DNA, and concentrations of 10^1^, 10^2^, 10^3^, 10^4^, 10^5^, 10^6^ copies/µL were used for the calibration curve.

**Table 1:** Primers used for real-time PCR analysis in the present study.

Genes	Sense primer (5′-3′)	Antisense primer (3′-5′)
Nestin	GATCGCTCAGATCCTGGAAG	GGTGTCTGCAAGCGAGAGTT
Sox2	AACGCCTTCATGGTATGGTC	TCTCGGTCTCGGACAAAAGT
HO-1	TGCTCGAATGAACACTCTGG	TCCTCTGTCAGCATCACCTG
xCT	AAACCCAAGTGGTTCAGACG	ATCTCAATCCTGGGCAGATG
CyclinD1	AGTGCGTGCAGAAGGAGATT	AGGAAGCGGTCCAGGTAGTT
Mash1	TCTCCTGGGAATGGACTTTG	GGTTGGCTGTCTGGTTTGTT
Math1	ACATCTCCCAGATCCCACAG	GGGCATTTGGTTGTCTCAGT
Math3	ACCCCGGGAAAGAGAATCTA	GCTCAGACCTTTGTCCATCC
Hes1	ACACCGGACAAACCAAAGAC	ATGCCGGGAGCTATCTTTCT
Hes5	GCAGCATAGAGCAGCTGAAG	AGGCTTTGCTGTGTTTCAGG
NeuroD1	ACGCAGAAGGCAAGGTGT	CCGCTCTCGCTGTATGATTT
OCTN1	GCCCCCTATTTCGTTTACCT	TGCATCTGCTCCAAGTTCTC
OCTN2	TCCGAACACGGAATATCAGG	ATGTCCCCATGCAAGTTAGG
OCTN3	GATCTGGTCCGAACACCAAA	AACAGCCGCCAGTAGGAAG
OCT1	GGCTCTGCCTGAGACTATTGA	TATGTGGGGATTTGCCTGTT
OCT2	CAATTTGCCGTGACTCTGC	GGGAGATCAACCATCTTGGA
OCT3	TTCGGCTGGCAGCTATATG	CTACGTCTTCCACCGTCTCC
MATE1	CCTGAATTCCGCTGTCTCTC	CAAAGCTTGTTGCTGGTTCA
PMAT	GGGATTACTGCCCAAGAGGT	GGAGCAGCAGCTTAGTGAGG
36B4	ACTGGTCTAGGACCCGAGAAG	TCCCACCTTGTCTCCAGTCT
GAPDH	AACTTTGGCATTGTGGAAGG	GGATGCAGGGATGATGTTCT

### Immunocytochemical analysis

Cortical NPCs or P19 cells were washed with PBS, then fixed with 4% paraformaldehyde for 20 min at 25°C and incubated for 30 min in blocking solution (3% bovine serum albumin and 0.2% Triton X-100 in PBS) at 25°C. They were then incubated overnight in 10-times-diluted blocking solution containing antiserum against OCTN1 (1∶500) or antibodies against nestin (1∶1000), βIII-tubulin (1∶1000), MAP2 (1∶1000), or GFAP (1∶1000) at 4°C, followed by washing with PBS and then reaction with Alexa Fluor series-conjugated secondary antibodies (1∶1000) for 1 h at 25°C. The cells were rinsed again with PBS, treated with mounting medium including DAPI, and observed under a LSM710 confocal laser scanning microscope.

### Uptake of [^3^H]ERGO in cultured cells

Cortical NPCs or P19 cells were washed with transport buffer (125 mM NaCl, 4.8 mM KCl, 1.2 mM CaCl_2_, 1.2 mM MgSO_4_, 1.2 mM KaH_2_PO_4_, 5.6 mM glucose and 25 mM HEPES, pH 7.4) and the uptake experiment was performed according to the silicone oil layer method [36] with some modifications. In brief, washed cells were incubated with 0.64 μM [^3^H]ERGO and an extracellular marker, 15 μM [^14^C]Mannitol, in transport buffer at 37°C for various periods in the presence or absence of 2–500 µM unlabeled ERGO. 200 μl aliquots of the mixture were withdrawn, and the cells were separated from the transport medium by centrifugal filtration through a layer of a mixture of silicon oil and liquid paraffin. Cells were then solubilized with 1.5 M KOH at 25°C overnight. The solubilized solution was neutralized with 5 M HCl, followed by addition of Clearsol I for liquid scintillation spectrometry. Protein concentration was determined with a Bio-Rad Protein Assay Kit. The uptake values were expressed as the cell-to-medium concentration (C/M) ratio (µl/mg of protein), obtained by dividing the uptake amount in the cells by the concentration of test compound in the medium and protein amount. Finally, C/M ratio of [^14^C]Mannitol, an extracellular marker, was subtracted from that of [^3^H]ERGO. Km values were calculated by fitting the data on dose-dependent inhibition by unlabeled ERGO to the Michaelis-Menten equation.

### Measurement of ERGO concentration

Liquid Chromatography-Mass Spectrometry/Mass Spectrometry (LC-MS/MS) analysis of ERGO was carried out on a LCMS-8040 (Shimadzu, Kyoto, Japan) equipped with HILIC column (phenomenex, 00F-4449-B0, 150×2 mm, 3 µm HILIC). Elution was preceded by means of a gradient with 0.3 mL/min flow rate using A solution (0.1% formic acid in water) and B solution (0.1% formic acid in acetonitrile). Initially, the proportion of B solution was sustained at 95% for 0.5 min and further linearly decreased to 30% within 3 min followed by a hold-time of 2 min. Subsequently, the proportion of B solution was increased to the initial conditions over 0.1 min followed by a hold-time of 2.4 min. The column temperature was kept at 50°C. The autosampler temperature was kept at 4°C. The injection volume was 1 µL. Analyses were performed on a LabSolutions instrument. MS–MS detection was performed in positive electrospray ionisation mode using multiple reaction monitoring (MRM) acquisition mode. The MRM was set at 230.3 to 127.0 m/z for ERGO, and 239.2 to 127.0 m/z for internal standard (L−(+)−Ergothioneine-d9). Nitrogen was used as nebulizer and argon was used as collision gas.

### Analysis of populations of neurons and astrocytes

Immunocytostaining visualized immature neuronal marker βIII-tubulin and mature neuronal marker MAP2 in green, and astrocyte marker GFAP in red. Quantification was performed by counting the number of cells immunoreactive for βIII-tubulin/MAP2 or GFAP in double immunocytochemical analysis, followed by calculation of the individual percentages versus the total number of cells stained with DAPI.

### Statistical analysis

All experiments were performed at least three times. Data are expressed as the mean ± S.E.M. The statistical significance of differences was determined by means of Student's *t*-test or one-way ANOVA with the Bonferroni/Dunn test and *p*<0.05 was regarded as denoting a significant difference.

## Results

### Functional expression of OCTN1

We previously reported that OCTN1 is functionally expressed in brain neurons and mouse neuroblastoma Neuro2a cells, which exhibit some characteristics of neuronal progenitor cells [27]. However, expression of OCTN1 in NPCs has not yet been clarified. So, we first examined gene expression of organic cation transporters in cultured cortical NPCs using a real-time PCR absolute quantification method. The expression level of OCTN1 mRNA was the highest among the cation transporters tested in the present study (Table 2). Interestingly, the expression level of OCTN1 mRNA was remarkably increased during 6 to 9 DIV, whereas it changed only minimally during 3 to 6 DIV (Table 2). Expression levels of OCTN2 and 3 mRNAs were lower than that of OCTN1. On the other hand, mRNAs for OCT1, OCT2, OCT3, MATE1, and PMAT were under the quantification limit (<60 copies/µg total RNA, Table 2).

**Table 2:** mRNA expression of organic cation transporters in mouse cultured cortical NPCs.^a)^

	3 DIV	6 DIV	9 DIV	Positive control^b)^
OCTN1	3475±919	4355±1202	11745±1994* #	321994±75129
OCTN2	168±9	100±66	102±39	35604±3133
OCTN3	648±77	587±326	609±128	1302±56
OCT1	<60	<60	<60	5596473±878051
OCT2	<60	<60	<60	110121±18322
OCT3	<60	<60	<60	1142±449
MATE1	<60	<60	<60	175261±36810
PMAT	<60	<60	<60	517±169
GAPDH	3.40±0.34×10^7^	4.68±0.63×10^7^	3.85±0.24×10^7^	N.D.^c)^

a) Copies/µg total RNA.

b) mRNA in testis for OCTN3, that in cerebral cortex for PMAT, and that in kidneys for other transporters.

c) N.D., not determined.

*P<0.05, significant difference from the corresponding value at 3 DIV.

# P<0.05, significant difference from the corresponding value at 6 DIV.

**Figure 1:**
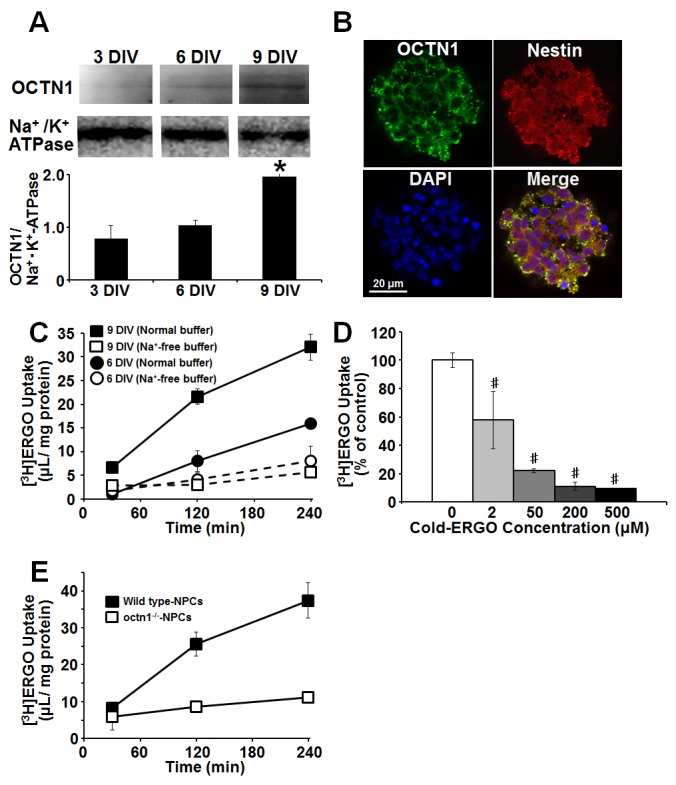
Functional expression of OCTN1 in mouse cultured cortical NPCs. (A) Membrane fraction was extracted by the centrifugation method, and the protein levels of OCTN1 in NPCs cultured for 3 to 9 DIV were determined by western blot analysis. Each value was normalized by the protein level of Na^+^/K^+^-ATPase. Each value represents the mean ± S.E.M. (n = 3). *P<0.05, significant difference from the value obtained in NPCs cultured for 3 DIV. (B) Cortical NPCs cultured for 9 DIV were fixed with 4% PA, followed by immunocytochemical detection of OCTN1 (green), NPC marker nestin (red) and nuclear marker DAPI (blue). The merged image is also shown. (C) Uptake of [^3^H]ERGO of cortical NPCs cultured for 6 and 9 DIV were measured at pH 7.4 and 37°C in the presence (closed) or absence of Na^+^ (open). Extracellular Na^+^ was replaced with *N*-methyl-D-glucamine at equimolar concentration. (D) Incubation was done with [^3^H]ERGO for 240 min in the presence or absence of various concentrations of unlabeled ERGO, followed by determination of [^3^H]ERGO uptake. (E) Cortical NPCs derived from wild-type or *octn1^−/−^* C57BL/6J embryonic mice were cultured for 9 DIV, and uptake of [^3^H]ERGO was measured at pH 7.4 and 37°C. Each value represents the mean ± S.E.M. (n = 3–6). ^#^P<0.05, significant difference from the control value obtained in NPCs incubated with [^3^H]ERGO alone.

In order to confirm expression of OCTN1 at the protein level, western blotting and immunocytochemical analysis were performed. Expression of OCTN1 at the protein level was also remarkably increased during 6 to 9 DIV (Fig. 1A). Immunoreactivity to OCTN1 antiserum was seen in NPC marker nestin-positive cells (Fig. 1B). Furthermore, to establish whether OCTN1 in NPCs is functional, uptake of the OCTN1 substrate ERGO was examined in cultured cortical NPCs derived from ICR embryonic mice. Uptake of [^3^H]ERGO into cortical NPCs cultured for 9 DIV increased markedly in a time-dependent manner, whereas uptake into NPCs cultured for 6 DIV increased only slightly. Uptake of [^3^H]ERGO at 9 DIV was 2-fold higher than that at 6 DIV (Fig. 1C), and this was compatible with the result of western blotting (Fig. 1A). In Na^+^-free buffer, [^3^H]ERGO uptake into NPCs cultured for both 6 and 9 DIV were much lower than that in normal transport buffer (Fig. 1C). The [^3^H]ERGO uptake at 9 DIV was inhibited by simultaneous addition of unlabeled ERGO in a dose-dependent manner over the concentration range of 2 to 500 µM (Fig. 1D). The Km value of 9.63 µM is close to the value of 4.68 µM obtained in human embryonic kidney 293 cells transfected with mouse OCTN1 [26]. In addition, [^3^H]ERGO uptake into NPCs derived from wild-type C57BL/6J embryonic mice was also observed as same as that from ICR embryonic mice, whereas NPCs derived from *octn1^−/−^* minimally incorporated [^3^H]ERGO (Fig. 1E).

### Suppression of cell proliferation by ERGO

**Figure 2:**
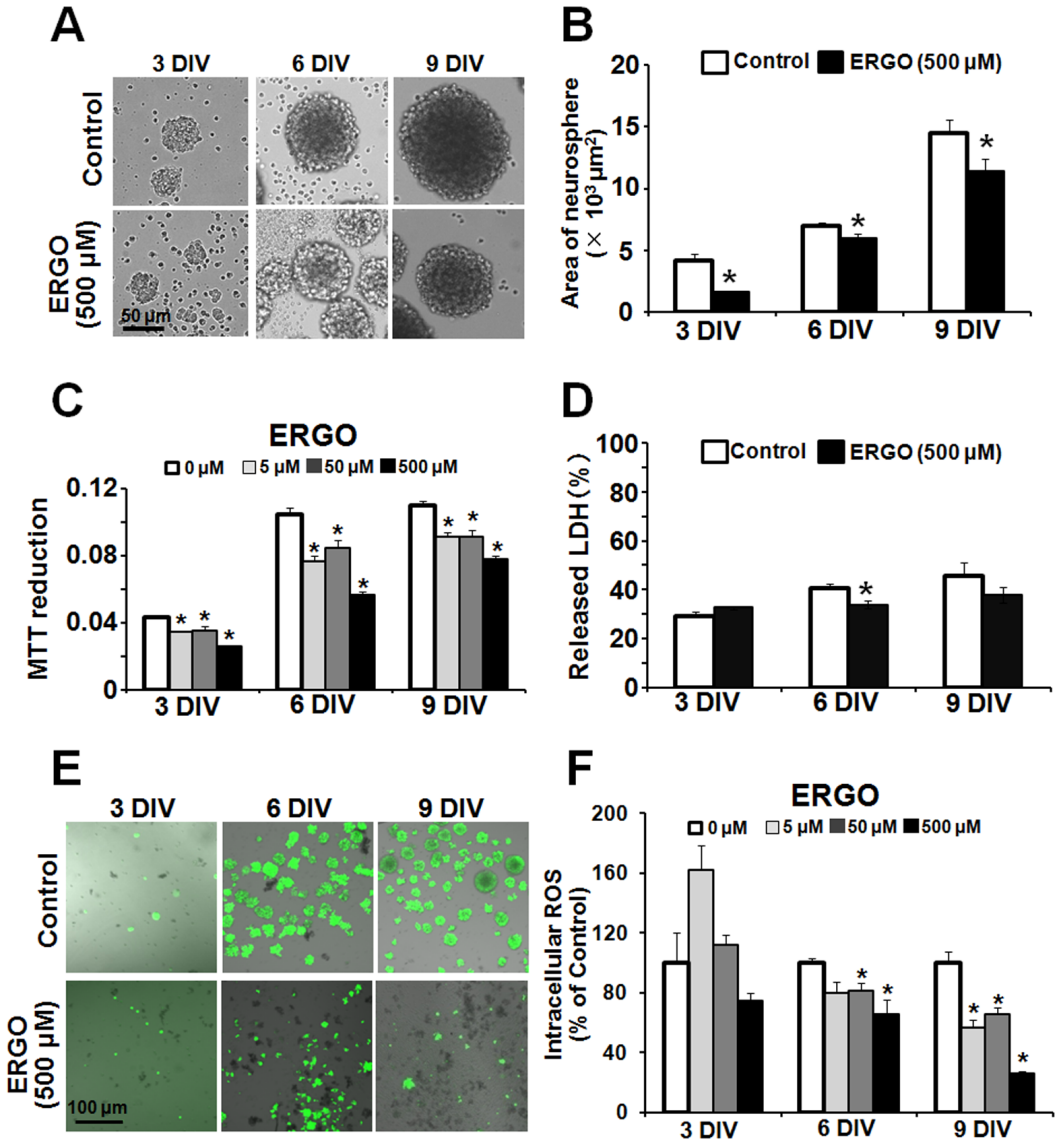
Effect of ERGO on proliferation of mouse cultured cortical NPCs. (A) NPCs were exposed to ERGO (500 µM) for 3 to 9 DIV, and (B) the area of neurospheres was quantified by using ImageJ. Each value represents the mean ± S.E.M. (n = 14). (C) Mitochondrial activity was measured by MTT assay in NPCs exposed to ERGO (0–500 μM) for 3 to 9 DIV. Each value represents the mean ± S.E.M. (n = 4). (D) Cytotoxicity was measured by LDH assay in NPCs exposed to ERGO (500 µM) for 3 to 9 DIV. Each value represents the mean ± S.E.M. (n = 3–4). (E) Intracellular ROS was determined by imaging with CM-H_2_DCFDA in NPCs exposed to ERGO (500 μM) for 3 to 9 DIV. (F) Intracellular ROS was quantified with ImageJ. The population of neurospheres showing a strong signal was measured with ImageJ. Each value represents the mean ± S.E.M. (n = 4). *P<0.05, significant difference from the control value.

To examine the physiological function of OCTN1 expressed in NPCs, ERGO was added to the culture medium of NPCs. OCTN1 substrate ERGO is an antioxidant, derived from the daily diet, and is present in blood and tissues of humans and other mammals [25], [37]. We speculated that ERGO might exert biological activities that would be regulated through its uptake via OCTN1 in NPCs. NPCs have two characteristics, self-renewal ability and pluripotentiality. We first examined the effect of ERGO on proliferation of NPCs. When ERGO (500 µM) was present in the culture medium up to 9 days, intracellular concentrations of ERGO in NPCs treated with ERGO for 6 and 9 days were 3572±629 and 10687±693 µg/g protein, respectively (Table 3), and neurospheres seemed to be smaller (Fig. 2A), so we quantified the area of neurospheres by using ImageJ software. Addition of ERGO to the culture medium significantly decreased the area of neurospheres regardless of the culture period (Fig. 2B). Moreover, MTT reduction (which reflects mitochondrial activity) was also decreased by ERGO in a dose-dependent manner (Fig. 2C), whereas release of LDH, which is a marker of cytotoxicity [34], was only minimally affected by ERGO (Fig. 2D). These results suggest that ERGO suppresses the proliferation of NPCs.

**Table 3:** Ergothioneine concentration in mouse cultured cortical NPCs exposed to ERGO (500 µM) for 6 and 9 days.

	Ergothioneine concentration (µg/g protein)^a)^
NPCs (6DIV, (−) ERGO)	<61
NPCs (6DIV, (+)ERGO)	3572±629 [0.0016 mol/L]^b)^
NPCs (9DIV, (−)ERGO)	<61
NPCs (9DIV, (+)ERGO)	10687±693# [0.0047 mol/L]^b)^

a) Concentration of ergothioneine was measured by LC-MS/MS.

b) The values shown in [ ] represent the mean values for intracellular concentration of ERGO estimated based on an assumption that cellular volume per g protein is 10 mL.

# P<0.01, significant difference from the corresponding value at 6 DIV.

**Figure 3:**
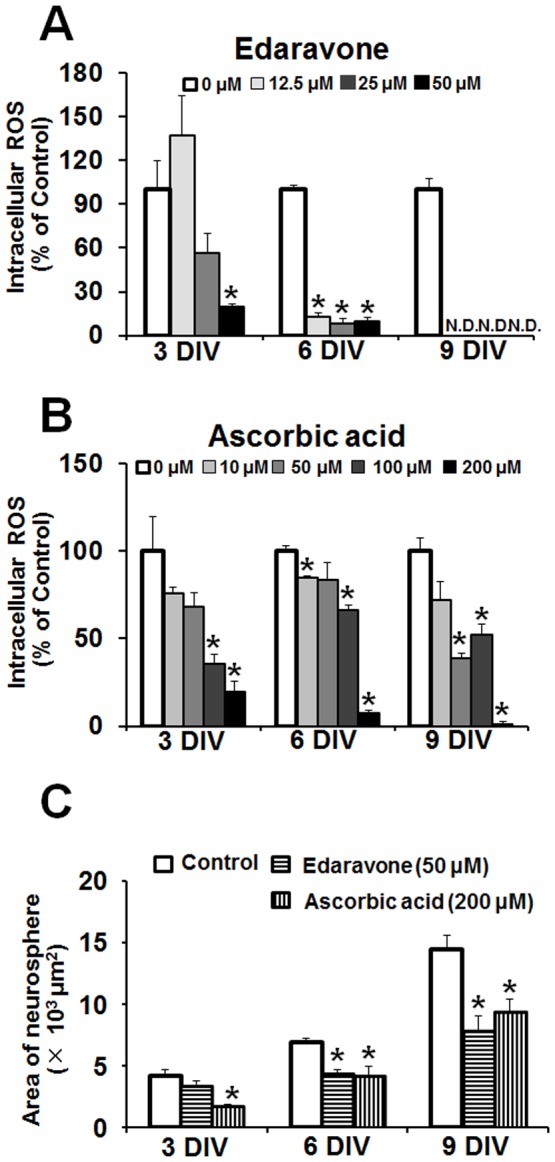
Comparison of proliferative activity between ERGO and other antioxidants. Intracellular ROS was determined by imaging with CM-H_2_DCFDA in NPCs exposed to edaravone (A) and ascorbic acid (B) for 3 to 9 DIV. Each value represents the mean ± S.E.M. (n = 3–4). In panel (C), the area of neurospheres was measured with ImageJ in NPCs cultured in either the presence or absence of edaravone (50 μM) or ascorbic acid (200 μM) for 3 to 9 DIV. *P<0.05, significant difference from the control value obtained in NPCs incubated in the absence of antioxidants. N.D., not detectable.

Then we examined whether the antioxidant activity of ERGO is involved in the suppression of cell proliferation by adding ERGO to the culture medium, because it has been reported that intracellular reactive oxygen species (ROS) promote proliferation of NPCs [30], [38]. Intracellular ROS was detected with ROS-sensitive fluorescent indicator CM-H_2_DCFDA as a green fluorescent signal (Fig. 2E). Addition of ERGO at the concentration of 500 µM seemed to decrease the number of neurospheres showing strong green fluorescence (Fig. 2E), concomitantly with inhibition of neurosphere formation (Fig. 2A). Quantitative analysis showed that ERGO significantly decreased the number of neurospheres showing green fluorescence at 6 and 9 DIV in a dose-dependent manner (Fig. 2F). Other antioxidants, edaravone (Fig. 3A) and ascorbic acid (Fig. 3B), also decreased the number of neurospheres showing green fluorescence in a concentration-dependent manner. Edaravone and ascorbic acid, as well as ERGO (Fig. 2B), also inhibited formation of neurospheres at the same concentrations that diminished intracellular ROS (Fig. 3C).

### Promotion of cell proliferation by knockdown of OCTN1

**Figure 4:**
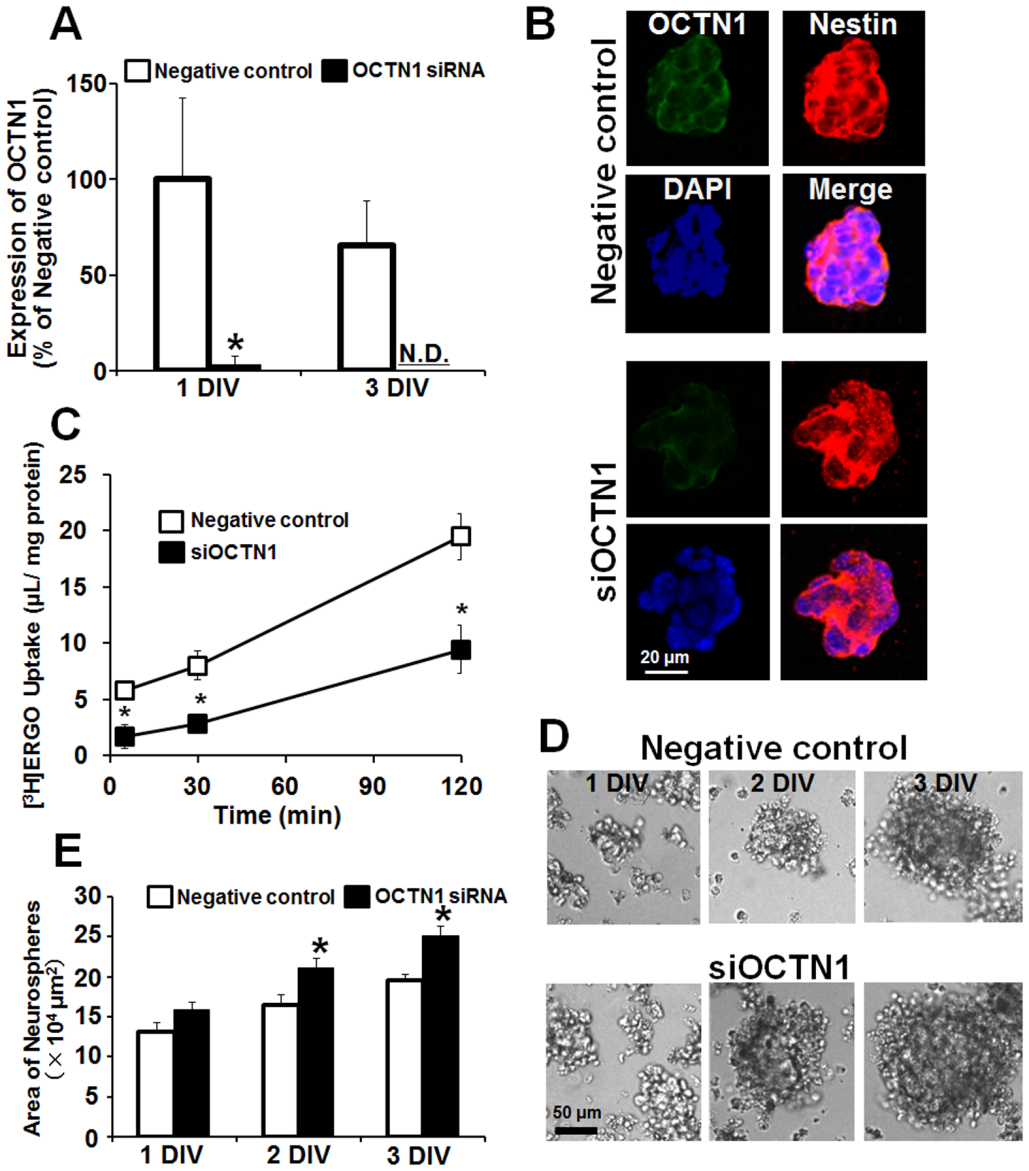
Effect of OCTN1 knockdown on proliferation of P19-NPCs. P19 cells were cultured in the presence of retinoic acid for 3-NPCs, and siRNA for OCTN1 or negative control siRNA was transiently transfected. (A) Total RNA was extracted for quantitative RT-PCR analysis of OCTN1 at 1 and 3 DIV after the transfection of control siRNA (white columns) and siOCTN1 (black columns). Each value is normalized by the expression level of GAPDH mRNA and represents the mean ± S.E.M. (n = 4–5). (B) P19-NPCs cultured for 3 DIV after the transfection were fixed with 4% PA, followed by immunocytochemical detection of OCTN1 (green), NPC marker nestin (red) and nuclear marker DAPI (blue). The merged image is also shown. (C) P19-NPCs were transiently transfected with siOCTN1 (black symbols) or negative control siRNA (white symbols), then cultured for 3 DIV, and the cells were incubated with [^3^H]ERGO at 37°C, followed by determination of [^3^H]ERGO uptake. Each value represents the mean ± S.E.M. (n = 3). (D) Phase-contrast micrographs were obtained for P19-NPCs cultured for 1 to 3 DIV after the transfection. (E) The area of neurospheres was quantified with ImageJ in P19-NPCs transfected with negative control siRNA (white columns) or siOCTN1 (black columns) and cultured for 1 to 3 DIV. *P<0.05, significant difference from the corresponding control value. N.D., not detectable.

To establish whether the effect of ERGO on proliferation of NPCs was mediated by OCTN1, we performed knockdown of OCTN1 in embryonic carcinoma P19 cells. We first measured the concentration of ERGO in FBS by LC-MS/MS to confirm that culture medium of P19 cells contains ERGO. FBS contained ERGO at 0.285±0.002 µg/mL (about 1.2 µM). The mRNA expression of OCNT1 was markedly and continuously reduced up to 3 DIV after the transfection of OCTN1 siRNA into P19-NPCs, compared to non-specific siRNA-transfected cells (Fig. 4A). Fig. 4B shows the localization of OCTN1 in P19-NPCs cultured for 3 DIV. Immunoreactivity to OCTN1 antiserum was seen on cell membrane of nestin-positive cells in the negative control group, but was greatly decreased in the siOCTN1-treated group (Fig. 4B). To confirm functional suppression of OCTN1, we investigated the transport activity of [^3^H]ERGO in siOCTN1-transfected P19-NPCs. Uptake of [^3^H]ERGO was markedly decreased in siOCTN1-transfected cells compared to cells transfected with negative control siRNA (Fig. 4C). Thus, the reduction in transport activity of [^3^H]ERGO was consistent with the knockdown of OCTN1 gene expression (Fig. 4A-C). The siOCTN1-treated group seemed to form larger neurospheres compared to the negative control (Fig. 4D). Quantitative analysis clearly showed that the area of neurospheres was increased in the siOCTN1-treated group compared to the negative control (Fig. 4E). These results suggest that transport of the antioxidant ERGO into cells via OCTN1 is involved in suppression of the proliferation of NPCs.

### Expression of proliferation-related genes

To further confirm whether exposure to ERGO and knockdown of OCTN1 regulate proliferation of NPCs via similar intracellular mechanisms, we used real-time PCR to examine the expression of several proliferation-related genes in cortical NPCs exposed to ERGO and P19-NPCs transfected with siOCTN1, focusing on heme oxygenase-1 (HO-1), cystine glutamate exchanger subunit (xCT), CyclinD1, and Sox2. HO-1 and xCT are induced as cellular antioxidant defense mechanisms in cells exposed to oxidative stress [39], [40]. CyclinD1 and Sox2 are required to maintain the self-renewal ability of NPCs [41], [42]. We also examined the expression of nestin, which is a marker of NPCs, in order to confirm that this property of NPCs was maintained.

**Figure 5:**
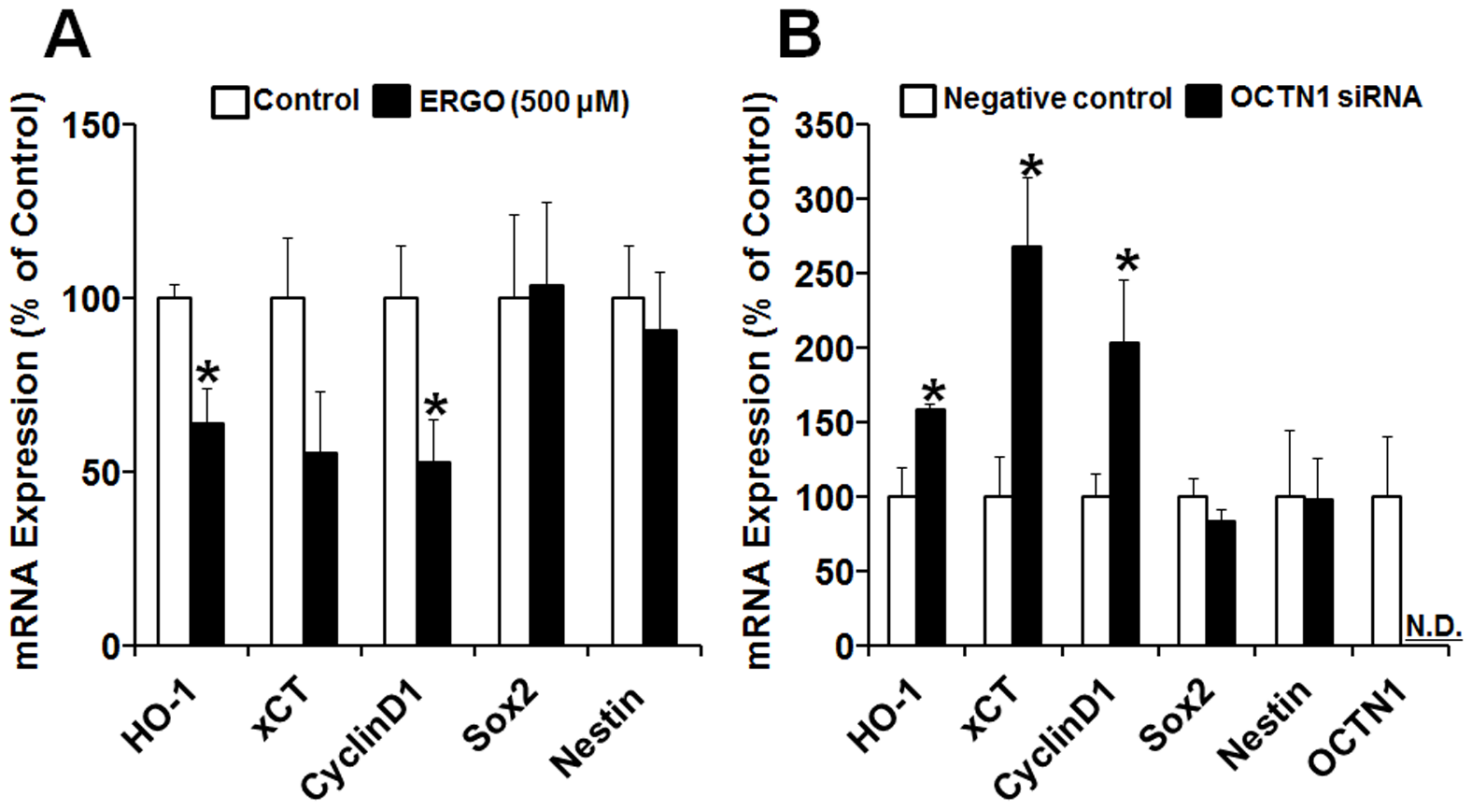
Effects of ERGO and OCTN1 knockdown on expression of proliferation-related genes in NPCs. (A) Mouse cultured cortical NPCs were exposed to ERGO (500 µM) for 9 DIV, and total RNA was extracted for quantitative RT-PCR analysis. Each value is normalized by the expression level of 36B4 mRNA and represents the mean ± S.E.M. (n = 5–6). *P<0.05, significant difference from the corresponding control value. (B) P19-NPCs were transfected with negative control siRNA (white columns) or siOCTN1 (black columns), and total RNA was extracted for quantitative RT-PCR analysis. Each value is normalized by the expression level of GAPDH mRNA and represents the mean ± S.E.M. (n = 3–8). *P<0.05, significant difference from the corresponding control value. N.D., not detectable.

Levels of mRNA for HO-1, xCT, and CyclinD1 in cortical NPCs were decreased or tended to be decreased by exposure to 500 µM ERGO (Fig. 5A), whereas they were significantly increased by transfection of siOCTN1 in P19-NPCs (Fig. 5B). Edaravone and ascorbic acid at concentrations that reduced intracellular ROS (50 and 200 µM, respectively) also attenuated mRNA expression of these three genes (data not shown). On the other hand, the expression levels of Sox2 and nestin were not changed by ERGO or siOCTN1 treatment (Fig. 5). These results indicate that exposure to ERGO and knockdown of OCTN1 regulate proliferation via similar intracellular mechanisms, suggesting that OCTN1-mediated ERGO transport may suppress proliferation of NPCs via a reduction of cellular oxidative stress.

### Promotion of neuronal differentiation by ERGO

**Figure 6:**
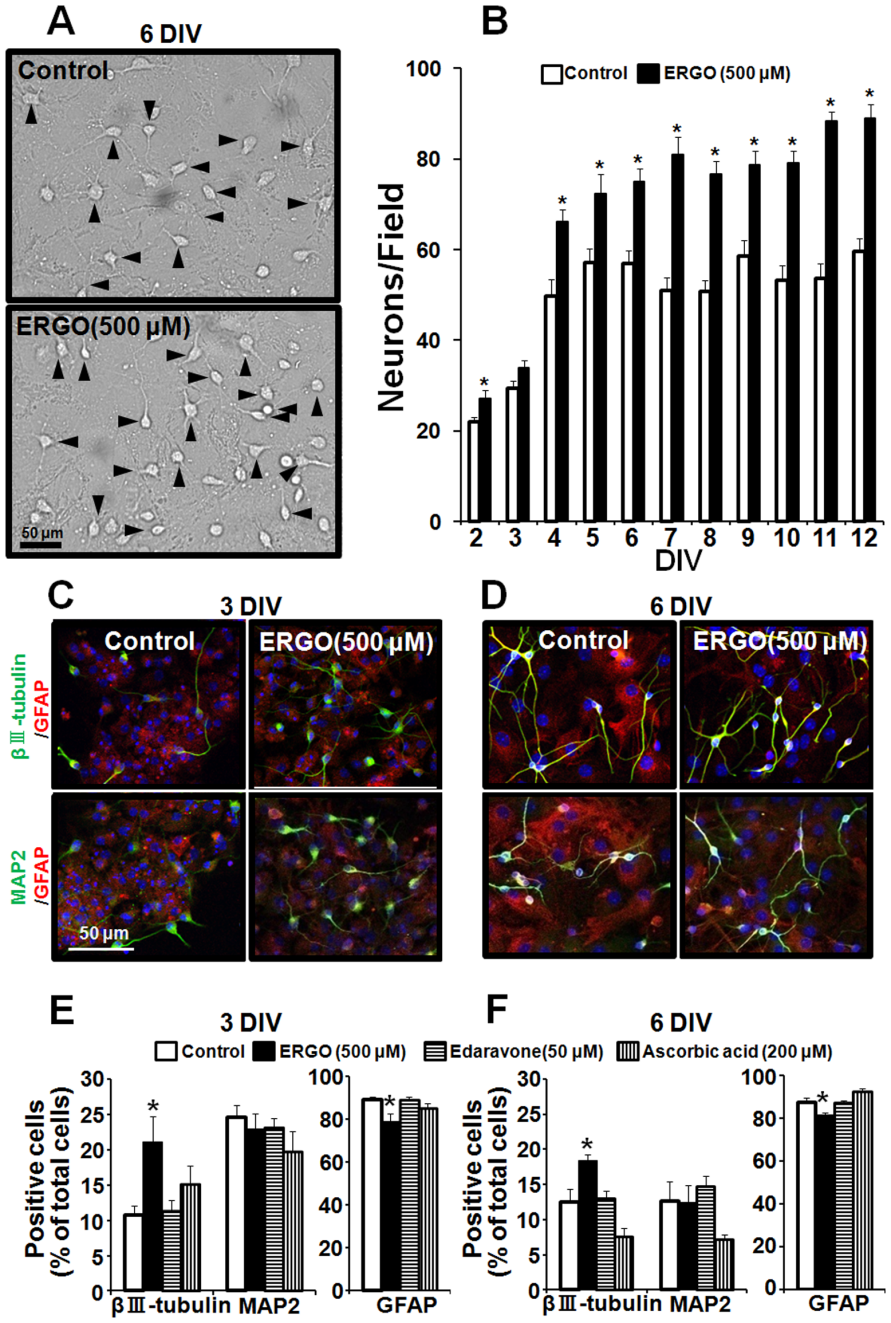
Effect of ERGO on differentiation in mouse cultured NPCs. NPCs were exposed to ERGO and other antioxidants for 9(A) Typical phase-contrast micrographs are shown. Black arrowheads represent differentiated neuron-like cells with neurites longer than the cell diameter. (B) The numbers of differentiated cells per field were counted for quantitative analysis in NPCs exposed to ERGO (black columns) or control (white columns). Each value represents the mean ± S.E.M. (n = 12). (C, D) NPCs exposed to ERGO (500 µM) for 9 DIV were induced to differentiate by adhesion culture for 3 or 6 DIV, and the cells were fixed with 4% PA, followed by immunocytochemical detection of immature neuronal marker βIII-tubulin (green), mature neuronal marker MAP2 (green), astroglial marker GFAP (red) and nuclear marker DAPI (blue). (E, F) NPCs exposed to each antioxidant for 9 DIV were induced to differentiate by adhesion culture for 3 or 6 DIV, and the numbers of cells positive for each marker were counted by using ImageJ and normalized by the number of DAPI-positive cells. Each value represents the mean ± S.E.M. (n = 6). *P<0.05, significant difference from the corresponding control value.

We then examined the effect of ERGO on neuronal differentiation of NPCs. Cortical NPCs were cultured in either the presence or absence of 500 μM ERGO, followed by induction of differentiation into neuronal or glial cells. Morphological observation indicated that neuron-like cells were increased in the ERGO-treated group compared to the control group (Fig. 6A). Quantitative analysis clearly showed that the number of neuron-like cells was continuously increased up to 12 DIV in the ERGO-treated group compared to the control group (Fig. 6B). We next performed immunocytochemical analysis. Immature neuronal marker βIII-tubulin and mature neuronal marker MAP2 were stained green, and astrocyte marker GFAP was stained red (Fig. 6C, D). Exposure to ERGO seemed to increase the number of βIII-tubulin-positive cells (Fig. 6C, D). Quantitative analysis showed that population of βIII-tubulin-positive cells was markedly increased, whereas that of GFAP-positive cells was significantly decreased at both 3 and 6 DIV in the ERGO-treated group compared to the control group (Fig. 6E, F). On the other hand, there was no significant difference in the number of MAP2-positive cells (Fig. 6E, F). Interestingly, other antioxidants, edaravone and ascorbic acid, did not affect the differentiation of NPCs (Fig. 6E, F).

### Suppression of neuronal differentiation by knockdown of OCTN1

**Figure 7:**
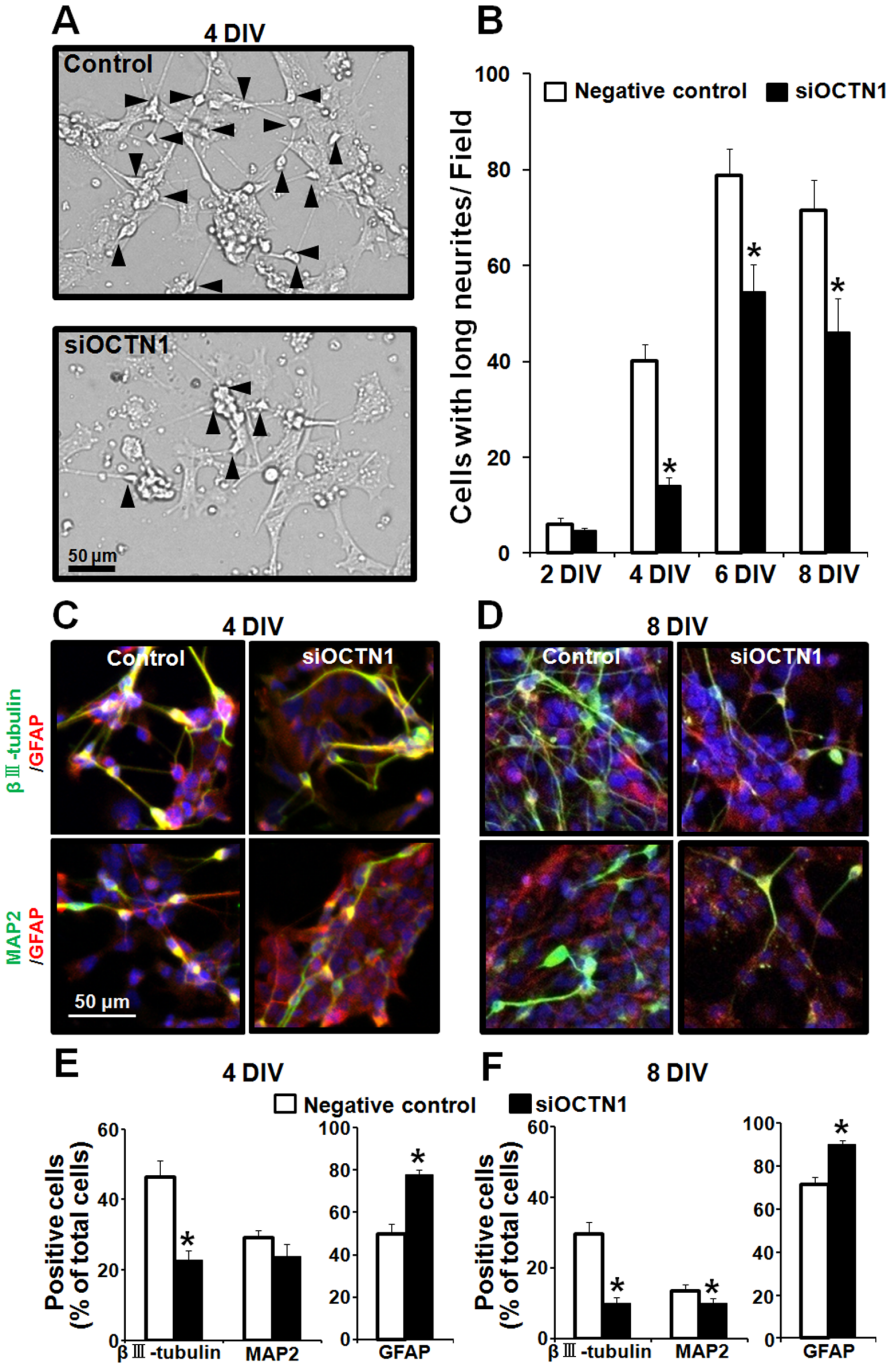
Effect of OCTN1 knockdown on differentiation of P19-NPCs. P19 cells were cultured in the presence of retinoic acid for 3-NPCs, and siRNA for OCTN1 or negative control siRNA was transiently transfected. Adhesion culture was started at 3 DIV after the transfection to induce differentiation into neurons and astrocytes. (A) Adhesion culture was maintained for 4 to 8 DIV, and typical phase-contrast micrographs are shown. Black arrowheads represent differentiated neuron-like cells with neurites longer than the cell diameter. (B) The numbers of differentiated cells per field were counted for 8 DIV in P19-NPCs after the transfection of siOCTN1 (black columns) or negative control siRNA (white columns). Each value represents the mean ± S.E.M. (n = 15). (C, D) P19-NPCs transfected with siOCTN1 or negative control were induced to differentiate by adhesion culture for 4 or 8 DIV, and then the cells were fixed with 4% PA, followed by immunocytochemical detection of immature neuronal marker βIII-tubulin (green), mature neuronal marker MAP2 (green), astroglial marker GFAP (red) and nuclear marker DAPI (blue). (E, F) The numbers of cells positive for each marker were counted by using ImageJ and normalized by the number of DAPI-positive cells. Each value represents the mean ± S.E.M. (n = 8). *P<0.05, significant difference from the corresponding control value.

To establish whether the effect of ERGO on neuronal differentiation of NPCs was mediated by OCTN1, we performed knockdown of OCTN1 in P19-NPCs. Neuron-like cells seemed to be decreased in the siOCTN1-treated group compared to the negative control (Fig. 7A). Quantitative analysis clearly showed that the number of neuron-like cells was continuously decreased up to 8 DIV in the siOCTN1-treated group compared to the control group (Fig. 7B). We next performed immunocytochemical analysis. Knockdown of OCTN1 seemed to decrease the number of βIII-tubulin- or MAP2-positive cells (Fig. 7C, D). Quantitative analysis showed that population of βIII-tubulin-positive cells was markedly decreased, whereas that of GFAP-positive cells was remarkably increased at both 4 and 8 DIV in the siOCTN1-treated group compared to the negative control group (Fig. 7E, F). In addition, the population of MAP2-positive cells was significantly decreased at 8 DIV in the siOCTN1-treated group (Fig. 7F). These results suggest that OCTN1 promotes differentiation of NPCs into neurons by mediating the transport of ERGO into cells.

### Expression of neuronal differentiation-related genes

To further confirm whether exposure to ERGO and knockdown of OCTN1 regulate differentiation of NPCs into neurons via similar intracellular mechanisms, we used real-time PCR to examine the expression of several neuronal differentiation-related genes in cortical NPCs exposed to ERGO and P19-NPCs transfected with siOCTN1, focusing on basic helix-loop-helix (bHLH), genes responsible for positive (Mash1, Math1, Math3 and NeuroD1) and negative (Hes1 and Hes5) regulation of neuronal differentiation of NPCs [14].

**Figure 8:**
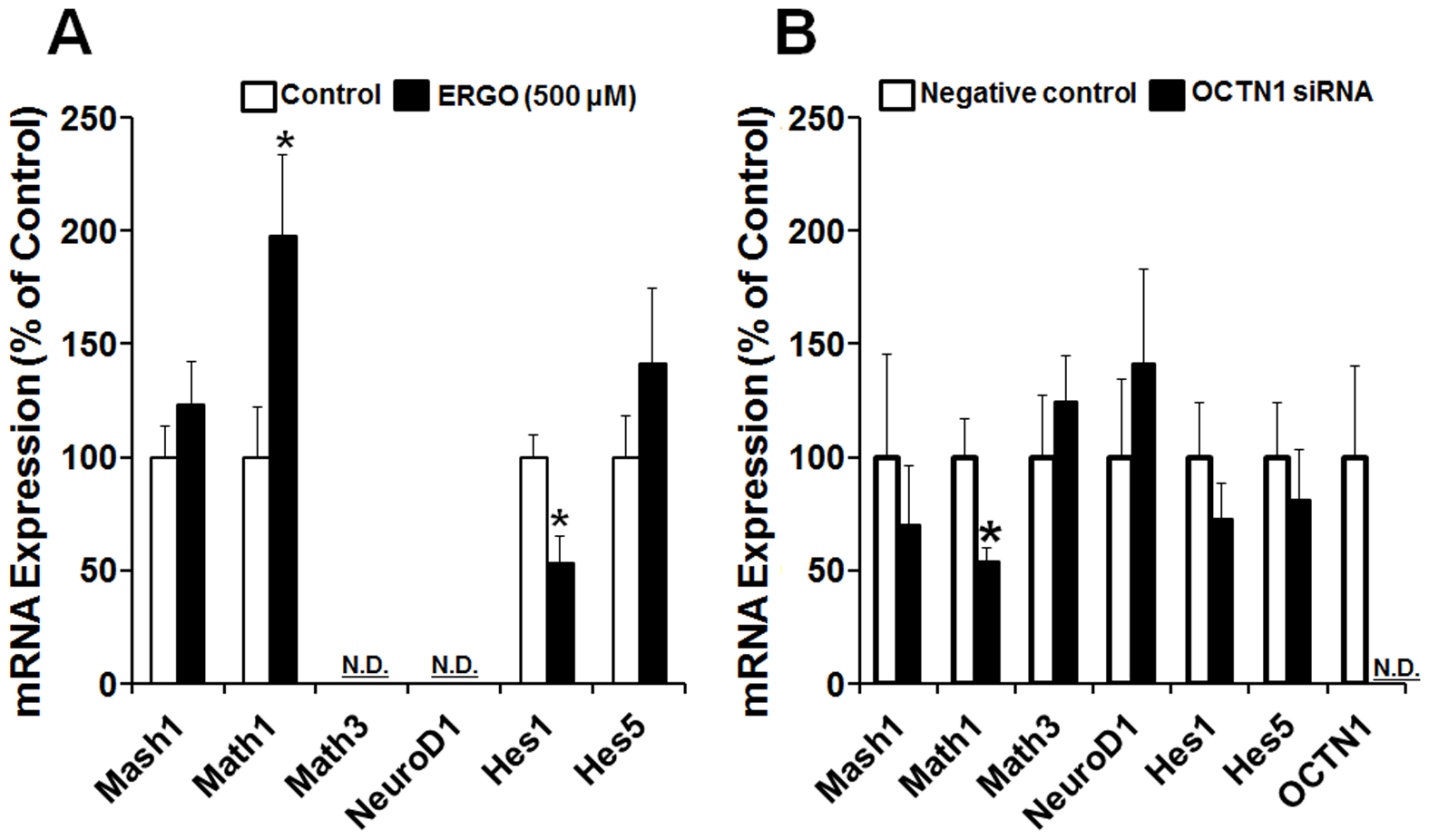
Effects of ERGO and OCTN1 knockdown on expression of neuronal differentiation-related genes in NPCs. (A) Mouse cultured cortical NPCs were exposed to 500 μM ERGO (black columns) or control (white columns) for 9 DIV, and total RNA was extracted for quantitative RT-PCR analysis. Each value is normalized by the expression level of 36B4 mRNA and represents the mean ± S.E.M. (n = 5–12). *P<0.05, significant difference from the corresponding control value. (B) P19-NPCs were transfected with negative control siRNA (white columns) or siOCTN1 (black columns), and total RNA was extracted for quantitative RT-PCR analysis. Each value is normalized by the expression level of GAPDH mRNA and represents the mean ± S.E.M. (n = 3–4). *P<0.05, significant difference from the corresponding control value. N.D., not detectable.

Expression of mRNA for Math1 was markedly increased by exposure to 500 µM ERGO in cortical NPCs (Fig. 8A), whereas it was significantly decreased by transfection of siOCTN1 in P19-NPCs (Fig. 8B). Other antioxidants (edaravone and ascorbic acid) at concentrations that diminished intracellular ROS (50 and 200 µM, respectively) did not affect the mRNA expression of Math1 (data not shown). On the other hand, Hes1 mRNA was clearly decreased, and Mash1 and Hes5 mRNAs were unchanged in cortical NPCs exposed to ERGO (Fig. 8A). Math3 and NeuroD1 mRNAs were not detected in cortical NPCs (Fig. 8A). The expression levels of Mash1, Math3, NeuroD1, Hes1 and Hes5 were unchanged by siOCTN1 treatment in P19-NPCs. These results suggest that OCTN1-mediated ERGO transport may promote differentiation of NPCs into neurons at least in part via up-regulation of Math1.

## Discussion

**Figure 9:**
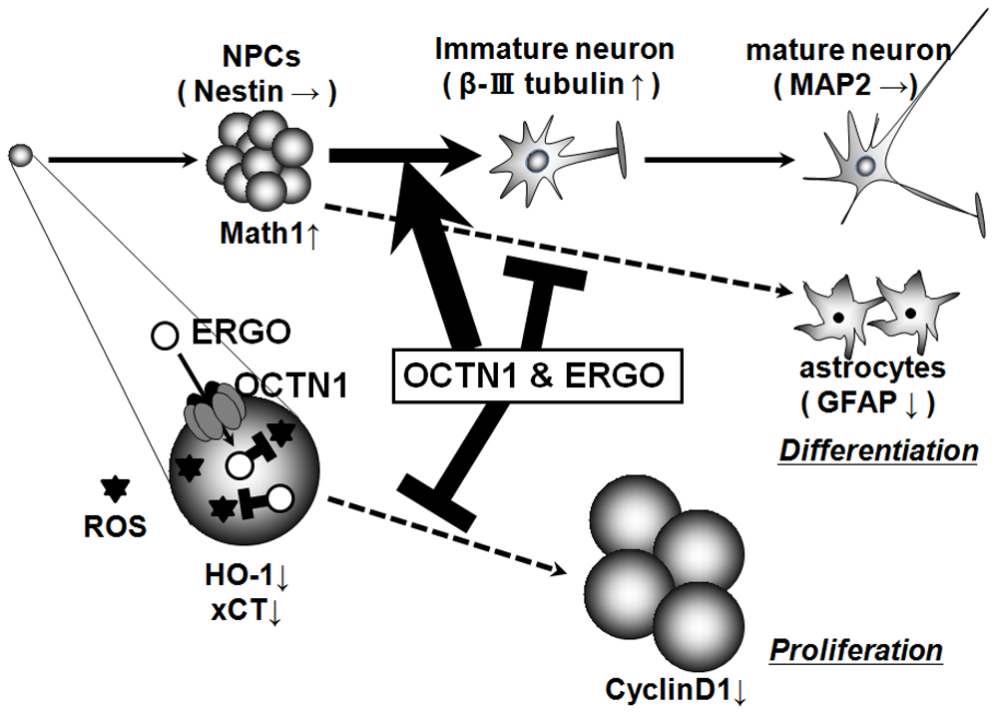
Involvements of OCTN1 and ERGO in proliferation and differentiation of NPCs. In NPCs, OCTN1-mediated ERGO transport inhibits cellular proliferation via the regulation of oxidative stress (Fig. 2–5), and promotes neuronal differentiation by modulating the expression of basic helix-loop-helix transcription factors via an unidentified action other than antioxidant activity (Fig. 6–8).

The present study is the first to demonstrate functional expression of OCTN1 in NPCs, and our results suggest that OCTN1 may promote neuronal differentiation and suppress proliferation of NPCs by transporting ERGO into the intracellular space (Fig. 9). The present findings may be relevant to the physiological regulation of NPCs *in vivo*, considering the everyday exposure of mammals to ERGO from ingested foods and the high concentration of food-derived ERGO present in the brain [25], [27]. Recently, it has been elucidated that proliferative ability and neuronal differentiation of NPCs are regulated by activation of various neurotransmitter receptors *in vitro*. Activation of dopamine D_3_, group I metabotropic glutamate (mGlu) or GABA receptors promoted proliferation of NPCs [43]–[45], whereas activation of NMDA, group III mGlu or α4β2 nicotinic acetylcholine (nACh) receptors suppressed it [46]–[49]. Activation of NMDA, GABA_B_, α4β2 nACh or M_2_ muscarinic acetylcholine receptors promoted neuronal differentiation of NPCs [45], [47], [49], [50], whereas activation of group III mGlu or GABA_A_ receptors suppressed it [45], [51]. However, the role of regulation by neurotransmitter receptors expressed in NPCs may be limited *in vivo*, because NPCs are undifferentiated cells that do not form synapses. On the other hand, several reports suggest that activation of neurotransmitter receptors may control physiological functions of NPCs *in vivo*. Systemic administration of NMDA decreased proliferation of NPCs in the adult murine dentate gyrus [52], whereas administration of a dopamine D_3_ agonist increased it in the adult murine subventricular zone [43]. Neurotransmitters may act directly in a paracrine or autocrine manner in NPCs, but control physiological functions of NPCs indirectly via their receptors expressed in other brain constituent cells. Transporters, on the other hand, are responsible for transport of physiological substrates between brain interstitial fluid and intracellular space, and therefore can directly control physiological functions of NPCs. So, transporters are also important candidates as intracellular environment regulatory molecules expressed on the cellular membrane of NPCs *in vivo*. This is exemplified by the present findings regarding the role of OCTN1 and ERGO (Fig. 9) in the regulation of NPCs.

According to the present gene expression analysis, OCTN1 may be the primary organic cation transporter in NPCs (Table 2). This would be consistent with a role of OCTN1 in the physiological regulation of NPCs. OCTN1 is classified as xenobiotic organic cation transporter. Several other such organic cation transporters, including OCT1-3, OCTN1-3, MATE1 and PMAT, are expressed in the body. These cation transporters each have characteristic patterns of expression and substrate recognition. OCT1, OCT2, OCT3, and OCTN3 are highly expressed in liver, kidney, ovary, and rodent testis, respectively [53]. MATE1 is an H^+^/cation antiporter and is highly expressed in kidney [54]. PMAT is highly expressed in human brain [55]. On the other hand, OCTN1 and OCTN2 are widely expressed in the body, and are essential for retention of ERGO and L-carnitine in the body, respectively [58]. In the present study, we have demonstrated that OCTN1 mRNA was expressed at a much higher level than OCTN2 and 3 mRNAs, while OCT1-3, MATE1 and PMAT mRNAs were below the quantification limit, in mouse cultured cortical NPCs (Table 2). These results imply a predominant role of OCTN1 in the physiological regulation of NPCs among these organic cation transporters.

Our results suggest that the antioxidant ERGO may suppress proliferation of NPCs via elimination of cellular oxidative stress. This idea is consistent with a previous finding that intracellular ROS promote the proliferation ability of NPCs [30], [38]. ERGO is a good substrate of OCTN1 *in vivo* and is well characterized as an antioxidant [59]. Indeed, ERGO decreased intracellular ROS similarly to other antioxidants, edaravone and ascorbic acid, in cortical NPCs (Fig. 2F, 3A, 3B). The addition of any of the three antioxidants to the culture medium of cortical NPCs inhibited formation of neurospheres (Fig. 2B, 3C) with concomitant down-regulation of HO-1 and xCT, which are markers of oxidative stress, and of CyclinD1, which is a marker of cellular proliferation (Fig. 5A). However, although 500 µM of ergothioneine did not affect the intracellular ROS generation at 3 DIV, it did exert marked proliferative effect. Some factors other than ROS are known to regulate proliferative activity in proliferative cells [60], [61]. From the results of ERGO exposure study (Fig. 6, 8), it is suggested that ERGO may have activity other than antioxidant effect, and this novel activity may not only promote neuronal differentiation of NPCs, but also suppress proliferation in NPCs. On the other hand, knockdown of OCTN1 greatly decreased ERGO transport activity (Fig. 4C) and promoted the formation of neurospheres (Fig. 4D, E) with concomitant up-regulation of HO-1, xCT, and CyclinD1 (Fig. 5B) in NPC-model P19 cells. By the way, the majority of uptake is maintained in NPCs transfected with siRNA for OCTN1. This is because mRNA expression of OCTN1 was perfectly decreased, but protein expression of OCTN1 might be remains on membrane. In fact, signal intensity of OCTN1 protein in the siOCTN1 treated group seemed to remain (Fig. 4B). In the knockdown study, FBS containing 0.285±0.002 µg/mL (about 1.2 µM) was added to the culture medium of P19 cells, and uptake of this ERGO was reduced by the siOCTN1 treatment. Thus, these results suggest that OCTN1-mediated ERGO transport may suppress proliferation of NPCs by reducing cellular oxidative stress (Fig. 9).

Among the three antioxidants, only ERGO promoted differentiation into neurons, while it suppressed differentiation into astrocytes in cortical NPCs (Fig. 6E, 6F), in contrast to the case of proliferative regulation, where all three antioxidants showed the same effect (Fig. 2B, 3C). In addition, only ERGO increased expression of Math1 and decreased that of Hes1 among the three antioxidants (Fig. 8A). These results suggest that ERGO may promote neuronal differentiation of NPCs through a mechanism(s) other than its antioxidant action. This idea is supported by a previous report that intracellular ROS did not affect the differentiation ability of NPCs [30]. On the other hand, knockdown of OCTN1 suppressed the rate of differentiation into neurons and promoted that into astrocytes (Fig. 7), with concomitant down-regulation of Math1 (Fig. 8B), in P19 cells. These results suggest that OCTN1-mediated ERGO transport may promote neuronal differentiation of NPCs concomitantly with up-regulation of Math1 (Fig. 9).

What is the mechanism through which ERGO promotes neuronal differentiation? Several amino acids, including glutamate and GABA, are well characterized as neurotransmitters, and ERGO also has an amino acid structure. Therefore, ERGO might act on amino acid receptors. Further, many researchers have reported that activation or inhibition of amino acid receptors influences the differentiation of NPCs into neurons [44]–[48], [51]–[52], [56], [57], [62]. ERGO might act as an amino acid receptor agonist or antagonist to promote differentiation of NPCs into neurons. However, if ERGO promoted neuronal differentiation via such membrane receptors, knockdown of OCTN1 would not be expected to decrease neuronal differentiation, because OCTN1 functions to promote intracellular accumulation of ERGO from the medium. Nevertheless, we found that OCTN1 knockdown did decrease neuronal differentiation (Fig. 7). These results can be explained in terms of the idea that intracellular ERGO imported through OCTN1 transport function induces expression of Math1 and promotes neuronal differentiation via an unidentified intracellular mechanism. Many intracellular mechanisms and signaling molecules are involved in differentiation of NPCs into neurons; for example, the G protein, Wnt, Notch, neurotrophin signaling cascades [63]–[66]. Intracellular ERGO might act on members of any of these signaling cascades, leading to the induction of transcription factors related to neuronal differentiation. Further studies are required to elucidate the mechanism underlying induction of neuronal differentiation via OCTN1-mediated ERGO transport.

In conclusion, OCTN1-mediated uptake of ERGO in NPCs inhibits cellular proliferation via regulation of oxidative stress, and also promotes cellular differentiation by modulating the expression of basic helix-loop-helix transcription factors via an unidentified mechanism different from antioxidant action.
